# Lymphatic invasion using D2-40 monoclonal antibody and its relationship to lymph node micrometastasis in pN0 gastric cancer

**DOI:** 10.1038/sj.bjc.6602739

**Published:** 2005-08-30

**Authors:** T Arigami, S Natsugoe, Y Uenosono, H Arima, Y Mataki, K Ehi, S Yanagida, S Ishigami, S Hokita, T Aikou

**Affiliations:** 1 Department of Surgical Oncology and Digestive Surgery, Field of Oncology, Course of Advanced Therapeutics, Kagoshima University Graduate School of Medical and Dental Sciences, 8-35-1 Sakuragaoka, Kagoshima 890-8520, Japan

**Keywords:** gastric cancer, lymph node micrometastasis, lymphatic invasion, reverse transcription–polymerase chain reaction, immunohistochemistry

## Abstract

The monoclonal antibody D2-40 is a specific lymphatic endothelial markers and D2-40 staining have been applicable to evaluate lymphatic invasion in various malignant neoplasms. In the present study, we investigated lymph node micrometastasis determined by immunohistochemistry (IHC) and reverse transcription–polymerase chain reaction (RT–PCR) in all dissected lymph nodes obtained from 80 patients with node-negative gastric cancer, and analysed the relationship between micrometastasis and clinicopathological findings including lymphatic invasion of the resected primary tumour using D2-40 immunohistochemical staining. The incidence of micrometastasis determined by IHC and RT–PCR was 11.3% (nine out of 80) and 31.3% (25 out of 80), respectively. Although haematoxylin–eosin (HE) staining revealed lymphatic invasion in 11.3% (nine out of 80) of patients, D2-40 staining uncovered new invasion in 23.8% (19 out of 80) of patients. In the diagnosis of HE and D2-40 staining, the incidence of micrometastasis was significantly higher in patients with lymphatic invasion than in those without lymphatic invasion (*P*=0.0150 and *P*<0.0001, respectively). Micrometastasis correlated more closely with D2-40 than with HE staining. We demonstrated a high incidence of micrometastasis and lymphatic invasion and a correlation between them even in pN0 gastric cancer. When planning less invasive treatment, the presence of such occult cancer cells should be considered.

Lymph node metastasis is one of the most important prognostic factors in patients with gastric cancer (Kwon and Kim, 1996; Nitti *et al*, 2003). Therefore, radical lymphadenectomy for gastric cancer has become a standard procedure, resulting in the long-term survival of patients with lymph node metastasis (Maehara *et al*, 1992). Nevertheless, several authors have reported that complete resection with radical lymphadenectomy that led to a node-negative (pN0) final diagnosis according to routine histological haematoxylin–eosin (HE) staining did not prevent recurrence (Maehara *et al*, 1996; Siewert *et al*, 1996; Ishida *et al*, 1997). The key causative factor of recurrent gastric cancer is lymph node micrometastasis. The sentinel node (SN) concept has recently been applied to gastrointestinal tract cancers including gastric cancer (Saha *et al*, 2000; Aikou *et al*, 2001; Uenosono *et al*, 2003). During sentinel node navigation surgery (SNNS), lymph node micrometastasis cannot be disregarded.

Lymph node micrometastasis can be detected because of sensitive biological methods such as real-time reverse transcription–polymerase chain reaction (RT–PCR). Some authors have proven that RT–PCR can detect lymph node micrometastasis in gastrointestinal tract or breast cancers (Mori *et al*, 1995; Matsumoto *et al*, 2002). Circulating cancer cells in the bone marrow or peripheral blood and free cancer cells in the peritoneal lavage fluid have been detected by RT–PCR (Gerhard *et al*, 1994; Burchill *et al*, 1995; Marutsuka *et al*, 2003). Thus, RT–PCR-based techniques can detect a minuscule number of occult cancer cells.

Although relationships between lymphatic invasion and lymph node micrometastasis in gastric cancer have been identified, the presence of lymphatic invasion has been based on the routine histological HE staining (Cai *et al*, 2000; Matsumoto *et al*, 2002). Since specific lymphatic endothelial markers have not been available, the accuracy of such findings has remained controversial. The D2-40 monoclonal antibody has been reported to recognise an oncofetal antigen present in fetal germ cells (Marks *et al*, 1999). And, this monoclonal antibody D2-40 can selectively detect lymphatic vessels (Kahn *et al*, 2002; Kahn and Marks, 2002; Fogt *et al*, 2004) as it can differentiate a fixation-resistant epitope on a 40 kDa *O*-linked sialoglycoprotein that is expressed in lymphatic endothelium but not in blood vessels (Kahn *et al*, 2002). That is, D2-40 staining is immunonegative in endothelial cells of blood and it have been applicable to evaluate the presence or absence of lymphatic invasion in various malignant neoplasms (Kahn and Marks, 2002). However, relationships between lymph node micrometastasis and clinicopathological findings have not been reported, including lymphatic invasion based on D2-40 immunohistochemical staining.

The purpose of the present study was to investigate the presence of lymph node micrometastasis using RT–PCR in patients who were diagnosed with pN0 gastric cancer by routine histological examination. In addition, we analysed the relationship between lymph node micrometastasis and clinicopathological findings, especially that of lymphatic invasion re-evaluated by monoclonal antibody D2-40 staining.

## MATERIALS AND METHODS

### Patients

We enrolled 80 patients who were diagnosed with pN0 gastric cancer according to routine histological examination with HE staining. All patients underwent curative gastrectomy with lymphadenectomy at the Department of Surgical Oncology and Digestive Surgery, Kagoshima University Hospital, between February 2003 and January 2005. None of the patients had received preoperative radiation therapy or chemotherapy. In all, 50 patients underwent distal gastrectomy, five proximal, 12 total and 13 partial gastrectomy. Based on the rules of the Japanese classification of gastric cancer (Japanese Gastric Cancer Association, 1998), the patients underwent D1 (*n*=21), D2 (*n*=6) or modified D2 (removal of all perigastric nodes and other nodes along the left gastric artery, common hepatic artery and celiac artery; *n*=53) lymphadenectomy. Table 1 shows the clinicopathological data of the 57 male and 23 female patients (age range, 41–84 years; average, 65 years) assessed according to the Japanese classification of gastric cancer (Japanese Gastric Cancer Association, 1998). The stomach was divided into U (upper), M (middle) and L (lower) thirds where 12, 45 and 23 tumours developed, respectively. The tumours were histopathologically classified as differentiated (papillary, well differentiated, moderately differentiated (tubular adenocarcinomas; *n*=39) or undifferentiated (poorly differentiated adenocarcinoma, mucinous adenocarcinoma and signet-ring cell carcinoma; *n*=41).

### Lymph nodes

We examined 1862 lymph nodes obtained from the 80 patients described above. The mean number of dissected lymph nodes was 23 (range, 2–69). Positive controls were lymph nodes from 10 patients whose gastric cancer was accompanied by histologically evident metastasis. Negative controls were lymph nodes from 15 patients without cancer (six gall bladder stones, four gastric adenomas, four gastric ulcers and one Crohn's disease). The lymph nodes were cut into 2 blocks at the plane of the largest dimension. Half of each lymph node was suspended in 1 ml of Isogen (Nippon Gene, Toyama, Japan) and immediately stored at −80°C. The remaining halves were fixed in 10% formaldehyde, embedded in paraffin, sliced into 3 *μ*m sections and stained with HE. Other sections were stained for immunohistochemistry (IHC) using cytokeratin (CK) monoclonal antibody. All specimens were collected from the patients after informed consent had been obtained in accordance with the institutional guidelines of our hospital.

### Immunohistochemistry

All lymph nodes were stained for IHC using a mouse monoclonal antibody against human CK AE1/AE3 (DAKO Corporation, Carpinteria, CA, USA). The sections were deparaffinised in xylene and rehydrated in ethanol, and then endogenous peroxidase activity was blocked by incubating the sections for 5 min in 3% hydrogen peroxide in methanol. The sections were then immersed in proteinase K (DAKO Corporation, Carpinteria, CA, USA) to activate the antigen and incubated with CK monoclonal antibody diluted 1 : 200 for 30 min. After two 5-min washes with phosphate-buffered saline (PBS), the reactions for CK were developed with an avidin–biotin complex immunoperoxidase technique (ABC method; VECTASTAIN ABC kit, Vector Laboratories, Inc., Burlingame, CA, USA). The sections were visualised using diaminobenzidine tetrahydrochloride. The negative control consisted of sections treated with the same protocol but with the primary antibody omitted. Normal gastric mucosa and the primary tumours of the specimens were used as positive controls and were consistently positive for CK.

The resected primary tumours were immunostained with D2-40 monoclonal antibody (DAKO Corporation, Carpinteria, CA, USA), fixed in 10% formalin and embedded in paraffin. Sectioned specimens on slides were deparaffinised in xylene and rehydrated with a graded series of ethanol. Endogenous peroxidase was blocked by immersion in methanol containing 3% hydrogen peroxide for 5 min. After a 5-min wash with PBS, the nonspecific binding was blocked in PBS containing 1% bovine serum albumin at room temperature for 30 min. The blocked sections were incubated at 4°C overnight with 50-fold diluted monoclonal antibody D2-40 in PBS, and D2-40 binding was visualised using an avidin–biotin complex immunoperoxidase procedure (Kahn *et al*, 2002; Kahn and Marks, 2002).

### Cell lines

MKN-45, an adenocarcinoma cell line that produces carcinoembryonic antigen (CEA) derived from a gastric cancer, was cultured in RPMI 1640 (Nissui Pharmaceutical Co., Ltd, Tokyo, Japan) supplemented with 10% fetal calf serum (Mitsubishi Kasei, Tokyo, Japan), 100 U ml^−1^ penicillin and 100 U ml^−1^ streptomycin.

### Real-time RT–PCR

Thawed lymph nodes were homogenised using FastPrep (Qbiogene, Inc., Carlsbad, CA, USA) and then total RNA extracted according to the manufacturer's instructions was dissolved in 20 *μ*l of water treated with diethylpyrocarbonate. The concentration, purity and amount of total RNA were determined by measuring absorption at 260 and 280 nm using a GeneQuant pro UV/Vis Spectrophotometer (Amersham Pharmacia Biotech, Cambridge, England). To avoid contamination with genomic DNA, 0.5 *μ*g of total RNA was digested for 15 min at 37°C with 1 U of DNase-I (Invitrogen, Life technologies, Foster City, CA, USA) that was subsequently inactivated by heating with 1 *μ*l of 25 mM ethylenediamine tetra-acetic acid (EDTA) at 65°C for 15 min. Complementary DNA (cDNA) was synthesised using the Advantage™ RT-for PCR Kit (Clontech Lab. Inc., Palo Alto, CA, USA) according to the manufacturer's protocol and then stored at −20°C. A CEA-specific oligonucleotide primer was designed based on that described by Gerhard *et al* (1994) as follows: sense, 5′-TGTCGGCATCATGATTGG-3′and antisense, 5′-GCAAATGCTTTAAGGAAGAAGC-3′. The donor and acceptor probe sequences for CEA identification were 5′-CCTGAAATGAAGAAACTACACCAGGGC-fluorescein and 5′-LC-Red640-GCTATATCAGAGCAACCCCAACCAGC-phosphorylation. CEA was amplified by PCR using a quantitative fluorescence LightCycler™ (Roche Diagnostics, Mannheim, Germany) in a 20 *μ*l reaction mixture containing 2 *μ*l of LightCycler™ FastStart DNA Master Hybridization Probes (Roche), 3.0 mM MgCl_2_, 0.5 *μ*M sense and antisense primers, 0.4 *μ*M fluorescent probe, 0.2 *μ*M LC-Red probe and 5 *μ*l of undiluted template cDNA in LightCycler™ capillaries (Roche). Before amplification, 0.32 *μ*l of anti-*Taq* DNA polymerase antibody (TaqStart™, Clontech Lab. Inc.) was incubated with the reaction mixture at room temperature for 5 min to avoid primer prolongation. The amplification profile consisted of one cycle at 95°C for 10 min (denaturation) followed by 35 cycles of 95°C for 10 s, 60°C for 15 s and 72°C for 5 s. Real-time PCR was monitored by measuring fluorescent signals at the end of the annealing phase for each cycle. The background signals were eliminated by setting the noise band in this study, and a sample was classified as positive if the intensity of fluorescence exceeded the noise band (Fit Points Method) (Marutsuka *et al*, 2003).

We quantified and confirmed the integrity of the RNA by comparison with real-time RT–PCR of the amplified glyceraldehyde-3-phosphatase dehydrogenase (GAPDH) housekeeping gene. The sense and antisense primers for GAPDH were 5′-TGAACGGGAAGCTCACTGG-3′ and TCCACCACCCTGTTGCTGTA-3′. The donor and acceptor probes for GAPDH were 5′-TCAACAGCGACACCCACTCCT-3′-fluorescein and 5′-LC-Red640-CACCTTTGACGCTGGGGCT-3′-phosphorylation. The GAPDH gene was amplified in 20 *μ*l of the same reaction mixture as described above in a LightCycler™ capillary (Roche). The amplification profile consisted of one cycle at 95°C for 10 min (denaturation) followed by 45 cycles of 95°C for 15 s, 60°C for 15 s and 72°C for 12 s. All primers and probes were synthesised and purified by reverse-phase high-performance liquid chromatography and the optimal reagent concentrations and PCR cycling conditions were established by the Nihon Gene Research Laboratories (Sendai, Japan). Each run of RT–PCR reaction included positive controls synthesised from MKN-45 cells, negative controls from RNA-negative samples. Real-time RT–PCR assays were repeated in triplicate. Quantification data were analysed using the LightCycler™ software (Roche).

We tested the sensitivity of the RT–PCR assay by spiking a series of 10-fold dilutions of MKN-45 cells (10^6^–10^0^) into 1 × 10^7^ peripheral blood mononuclear cells (PBMCs) from a normal healthy volunteer who did not express CEA mRNA. Total RNA extracted as described above was assayed by real-time RT–PCR. The RT–PCR product was identified by 2% agarose-gel electrophoresis in Tris-acetate EDTA buffer and visualised after staining with ethidium bromide.

### Statistical analysis

All statistical calculations were performed using StatView statistical software version 5.5 (SAS Institute, Cary, NC, USA). All data were statistically compared using the *χ*^2^ test. A *P*-value of <0.05 was considered statistically significant.

## RESULTS

### Sensitivity of RT–PCR and CEA mRNA expression in control samples

**Figure 1:**
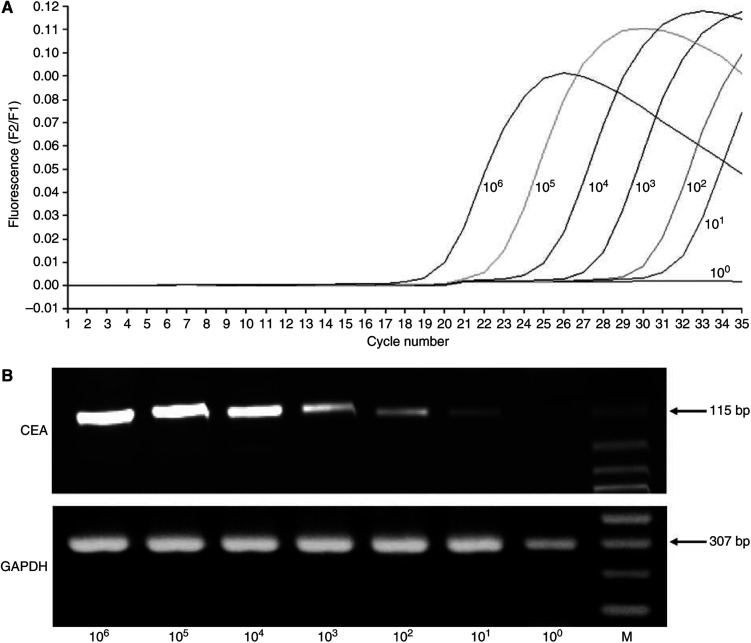
Sensitivity of real-time RT–PCR using CEA mRNA as the primer. (**A**) The intensity of fluorescence *vs* PCR cycles. (**B**) Ethidium bromide-stained agarose gels following electrophoresis of CEA and GAPDH RT–PCR products. M=DNA molecular weight marker.

CEA mRNA was detectable in MKN-45 cancer cell lines at concentrations as low as 10^1^ tumour cells per 10^7^ PBMCs (Figure 1). Although 10 lymph nodes from 10 patients with gastric cancer accompanied by histologically evident metastasis expressed CEA mRNA (positive control), none of 15 lymph nodes from 15 patients without cancer expressed CEA mRNA under the same conditions (negative control, Table 2).

### Incidence of lymph node micrometastasis by IHC and RT–PCR

Lymph node micrometastasis was identified in nine of the 80 patients (11.3%) and in 34 of the 1862 nodes (1.8%) by IHC, whereas RT–PCR revealed lymph node micrometastasis in 25 patients (31.3%) and 66 nodes (3.5%). Of these 66 lymph node micrometastases, 33 were detected only by RT–PCR. On the other hand, only one lymph node micrometastasis was detected by IHC alone.

### Diagnostic comparison of lymphatic invasion determined by HE and D2-40 staining

**Figure 2:**
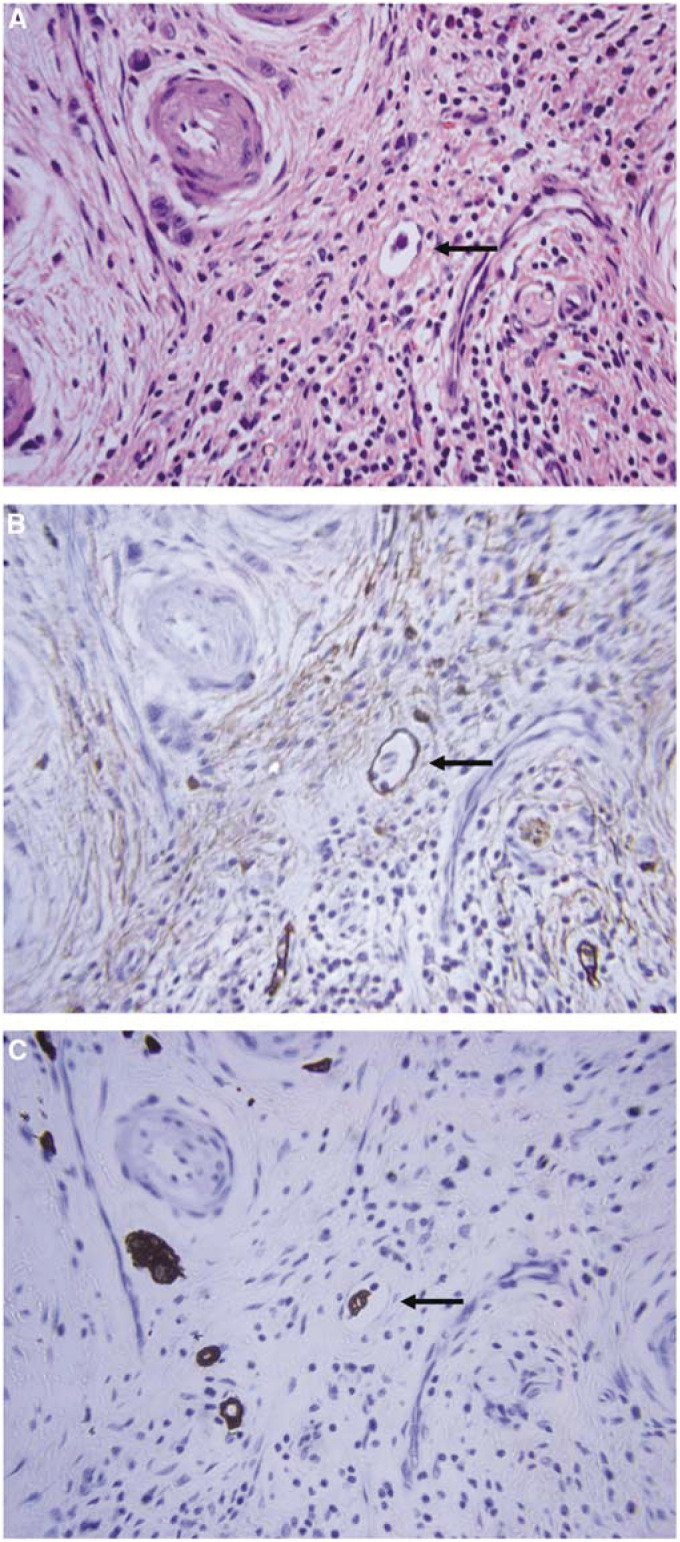
Example of a patient diagnosed as being free of lymphatic invasion by routine histological examination. (**A**) Routine haematoxylin–eosin staining. (**B**) D2-40 staining. (**C**) Cytokeratin (AE1/AE3) staining. Original magnification × 400.

**Figure 3:**
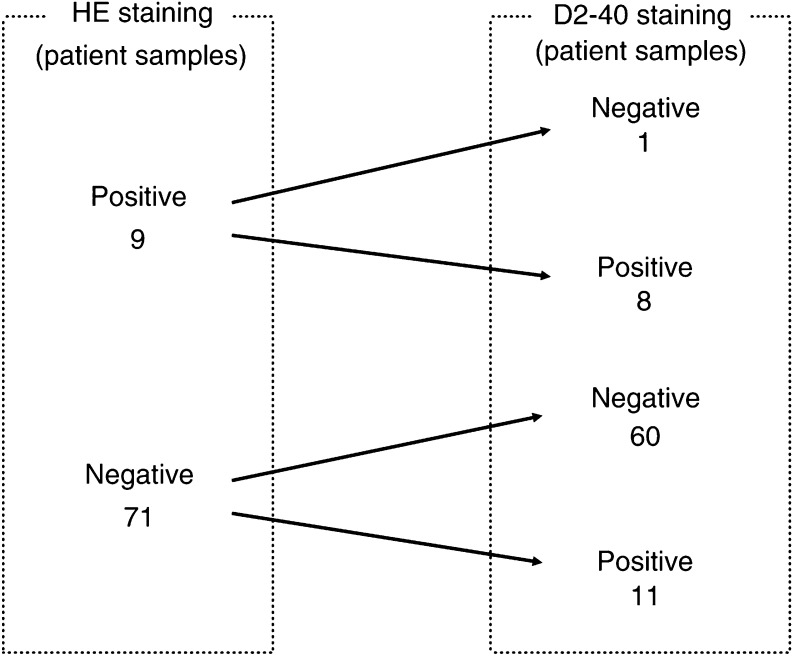
Diagnostic comparison of lymphatic invasion between haematoxylin–eosin (HE) and D2-40 staining in 80 patients.

Lymphatic vessels were clearly delineated by D2-40 staining (Figure 2). The patient described in this figure had a pT2 tumour that routine histological examination had determined was pN0 and free of lymphatic invasion (Figure 2A). However, D2-40 staining revealed obvious lymphatic invasion (Figure 2B). A single cancer cell was identified by CK immunohistochemical staining in this lymphatic vessel (Figure 2C). Some lymphatic vessels that were HE negative were obviously positive according to D2-40 staining (Figure 2). Histological HE staining revealed lymphatic invasion from the primary tumour in nine of the 80 patients (11.3%, Figure 3). In eight of these nine, lymphatic invasion detected by HE staining was in accord with the results of D2-40 staining. On the other hand, lymphatic invasion was newly detected in 11 (13.8%) patients who were diagnosed as free of lymphatic invasion by HE staining. Thus, the incidence of lymphatic invasion increased from 11.3% by HE staining to 23.8% by D2-40 staining.

### Correlation between lymph node micrometastasis and clinicopathological findings of patients with gastric cancer

With regard to the depth of tumour invasion, lymph node micrometastasis was identified in 20 of 74 (27.0%) and in five of six (83.3%) patients with pT1 with pT2 tumours, respectively. The incidence of lymph node micrometastasis significantly differed between pT1 and pT2 tumours even though fewer patients had pT2 tumours (*P*=0.0042). However, significant difference was not found in tumour location, histological type and venous invasion (Table 3). Both HE (*P*=0.0150) and D2-40 immunohistochemical staining (*P*<0.0001) found a significantly higher incidence of lymph node micrometastasis in patients with, than without lymphatic invasion. However, lymph node micrometastasis more closely correlated with D2-40 than with HE staining (Table 4).

## DISCUSSION

Increasingly sensitive immunohistochemical and biological techniques have recently led to the detection of lymph node micrometastasis. Reports indicate that RT–PCR can detect lymph node micrometastasis more sensitively than IHC (Mori *et al*, 1995; Matsumoto *et al*, 2002). Here, we found lymph node micrometastasis in 11.3% of patients with pN0 gastric cancer according to IHC, and in 31.3% according to RT–PCR. This means that the lymphatic spread of occult cancer cells is beyond the limits of detection by routine histological examination. Several reports have described a relationship between lymph node micrometastasis and clinicopathological characteristics in gastric cancer (Cai *et al*, 2000; Matsumoto *et al*, 2002). Matsumoto *et al* (2002) reported that the incidence of lymph node micrometastasis is significantly higher in pN0 patients with lymphatic invasion than in those without lymphatic invasion. However, lymphatic invasion was evaluated only by conventional HE staining in these reports.

In the present study, the majority of patients (92.5%) had early gastric tumour and they underwent the standard lymphadenectomy. None of 80 enrolled patients died or recurred because of short follow-up period within 2 years. Therefore, we could not find the significant difference in survival rate according to the presence or absence of lymph node micrometastasis. However, we think that meticulous follow-up examination should be needed in patients with lymph node micrometastasis for long period.

We used D2-40 staining to identify lymphatic vessels in the present study. Kahn and Marks (2002) reported that D2-40 antibody could be useful to ascertain the presence or absence of lymphatic invasion in various malignant neoplasms. They reported that the false negative and false positive rates of HE staining in breast cancer are 18 and 4%, respectively. Similarly, we found higher detection rates with D2-40, compared with HE staining (23.8 *vs* 11.3%). D2-40 staining newly revealed lymphatic invasion in 11 of 71 patients (15.5%) in whom HE staining was negative. These results indicated that lymphatic invasion could be present in some patients who have been diagnosed as free of lymphatic invasion by routine histological examination. Thus, since diagnosis of lymphatic invasion was clearly enhanced by D2-40 staining, it is necessary to examine lymphatic invasion by D2-40 staining for accurate diagnosis, especially in early gastric cancer.

D2-40 staining indicated that the incidence of lymph node micrometastasis was significantly higher in patients with, than without lymphatic invasion (*P*<0.0001). This finding demonstrated that lymph node micrometastasis, which is the initial stage of lymph node metastasis, is closely related to lymphatic invasion.

Early gastric cancer has recently been treated by endoscopic mucosal resection (EMR) (Takekoshi *et al*, 1994). However, a disadvantage of this approach is that imaging cannot accurately diagnose lymph node micrometastasis. The clinical significance of lymph node micrometastasis in gastric cancers remains controversial (Ishida *et al*, 1997). Therefore, lymphatic invasion in resected specimens should be examined not only by staining with HE but also with D2-40 to predict lymph node micrometastasis.

The SN concept was originally advocated by Morton *et al* (1992) to treat patients with melanoma. According to this concept, SN is the first lymph node to receive lymphatic flow from the primary tumour, and micrometastasis develops at this site. Lymph node dissection areas can be accurately assessed by SNNS in patients with breast cancer and malignant melanoma (Veronesi *et al*, 1997; Edwards *et al*, 1998). The SN concept has recently been applied to gastrointestinal tract cancers including gastric cancers (Saha *et al*, 2000; Aikou *et al*, 2001; Uenosono *et al*, 2003), but its clinical application remains controversial. An assured diagnosis of lymph node micrometastasis determined by RT–PCR is essential when performing SNNS, since the clinical significance of lymph node micrometastasis is also contentious (Ishida *et al*, 1997). It is difficult to routinely assess micrometastasis in all dissected lymph nodes using IHC and RT–PCR in the aspects of time consuming and cost for practical use. Therefore, we should select the cases in which the diagnosis of lymph node micrometastasis reflects the operative procedure. Actually, we think that an intraoperative diagnosis of micrometastasis is essential in SNNS. If SNNS becomes acceptable for patients with gastric cancer in the near future, then minimally invasive surgery with personalised lymphadenectomy might be safely performed in consideration of lymph node micrometastasis.

In conclusion, we demonstrated that RT–PCR can sensitively detect lymph node micrometastasis, and that D2-40 staining can identify lymphatic invasion at a higher frequency than routine histological HE staining. Lymph node micrometastasis, which is the initial stage of lymph node metastasis, was closely related to lymphatic invasion. Thus, information about micrometastasis and lymphatic invasion obtained by RT–PCR and D2-40 staining will be useful for considering less invasive treatment strategies such as EMR or SNNS.

## Figures and Tables

**Table 1:** Clinicopathological findings of 80 patients with gastric cancer

*Gender*	
Male	57
Female	23
	
*Tumour location*	
Upper	12
Middle	45
Lower	23
	
*Histological type*	
Differentiated	39
Undifferentiated	41
	
*Depth of tumour invasion*	
pT1	74
pT2	6
	
*Lymphatic invasion* a	
Negative	71
Positive	9
	
*Venous invasion* a	
Negative	73
Positive	7

pT1=invasion of mucosa or submucosa; pT2=invasion of muscularis propria or subserosa.

a Lymphatic and venous invasion were identified based on haematoxylin–eosin staining.

**Table 2:** Expression of CEA mRNA in lymph nodes from patients with and without gastric cancer

	**Expression of CEA mRNA**		
**Lymph nodes**	**Negative**	**Positive**	**Total**
Metastatic lymph nodes of gastric cancer^a^	0	10	10
Lymph nodes of benign disease	15	0	15

a These lymph nodes were diagnosed with evident metastases by haematoxylin–eosin staining.

**Table 3:** Correlation between lymph node micrometastasis and clinicopathological findings

	**Micrometastasis (%)**		
**Variables**	**−(*n*=55)**	**+(*n*=25)**	***P*-value**
*Tumour location*			
Upper	10 (83.3)	2 (16.7)	0.4935
Middle	30 (66.7)	15 (33.3)	
Lower	15 (65.2)	8 (34.8)	
			
*Histological type*			
Differentiated	28 (71.8)	11 (28.2)	0.5666
Undifferentiated	27 (65.9)	14 (34.1)	
			
*Depth of tumour invasion*			
pT1	54 (73.0)	20 (27.0)	0.0042
pT2	1 (16.7)	5 (83.3)	
			
*Venous invasion* a			
Negative	52 (71.2)	21 (28.8)	0.1218
Positive	3 (42.9)	4 (57.1)	

pT1=invasion of mucosa or submucosa; pT2=invasion of muscularis propria or subserosa.

a Venous invasion was identified based on haematoxylin–eosin staining.

**Table 4:** Correlation between lymph node micrometastasis and lymphatic invasion

	**Micrometastasis (%)**		
**Lymphatic invasion**	**−(*n*=55)**	**+(*n*=25)**	***P*-value**
*HE staining*			
Negative	52 (73.2)	19 (26.8)	0.0150
Positive	3 (33.3)	6 (66.7)	
			
D2-40 staining			
Negative	52 (85.2)	9 (14.8)	<0.0001
Positive	3 (15.8)	16 (84.2)	

HE=haematoxylin–eosin.
